# Spatial Patterns of Species Diversity of Amphibians in a Nature Reserve in Eastern China

**DOI:** 10.3390/biology12030461

**Published:** 2023-03-16

**Authors:** Yanmei Wang, Huali Hu, Lei Feng, Jingyi Chen, Junjie Zhong, Rachel Wan Xin Seah, Guohua Ding

**Affiliations:** 1 Laboratory of Amphibian Diversity Investigation, College of Ecology, Lishui University, Lishui 323000, China; 2 College of Biology and the Environment, Nanjing Forestry University, Nanjing 210037, China; 3 Department of Biological Science, National University of Singapore, Singapore 117558, Singapore

**Keywords:** amphibian, elevation, phylogenetic diversity, spatial pattern, taxonomic diversity, Rapoport’s rule

## Abstract

**Simple Summary:**

Montane habitats are characterized by mountainous regions with steep environmental gradients within a small geographic area. These habitats play a prominent role in shaping vertical species distributions due to multiple correlations between elevation and a host of environmental factors, such as temperature, humidity, and anthropogenic disturbances. Thus, they make for a fascinating natural laboratory for biodiversity studies. Our study addresses the regional and elevational patterns of taxonomic and phylogenetic species diversity in the amphibians of Fujian Junzifeng National Nature Reserve located in eastern China. By combining various diversity indices, we provide a multi-dimensional framework to study the distribution mechanism of amphibian species diversity along a large elevational gradient, in the hopes of contributing to a greater understanding and effective conservation of mountainous amphibian diversity.

**Abstract:**

Elevational gradients provide an excellent opportunity to assess biodiversity patterns and community structure. Previous studies mainly focus on higher elevations or are limited to small areas in mountainous regions. Little information can be found on amphibian biodiversity in middle- and low-elevational areas, hence our study was devoted to filling up the current gaps in these research areas. To understand the variability of biodiversity of amphibian species in the Fujian Junzifeng National Nature Reserve in eastern China, our study included taxonomic and phylogenetic components to describe the various patterns of regional and elevational distribution. The results showed that (1) most of the taxonomic and phylogenetic diversity metrics were correlated; with regard to the surveyed area, Faith’s phylogenetic diversity index (*PD*) and net relatedness index (*NRI*) were positively correlated with the Shannon–Wiener index (*H’*), Margalef index (*DMG*), and species richness (*S*), while negatively with the Pielou index; whereas for elevation, only the Pielou index was positively correlated with the nearest taxon index (*NTI*), but negatively with other indices; (2) taxonomic and phylogenetic diversities did not differ among the three survey locations but differed significantly along the elevational gradient; Simpson index, *H’*, *S*, and *DMG* had a hump-shaped relationship with elevations, and *PD* decreased gradually with the increase in elevation, whereas *NRI* and *NTI* sharply increased at the elevation above 900 m; (3) the species range size and the corresponding midpoint of amphibians were affected by a strong phylogenetic signal, which supports the elevational Rapoport’s rule upon removal of *Pachytriton brevipes* and *Boulenophrys sanmingensis* from the study.

## 1. Introduction

The key mechanisms shaping the distribution of biodiversity are crucial in macroecology and conservation biogeography [1]. This is especially true for montane regions; these areas are home to many endemic and threatened species within a small geographic range which makes them highly prioritized for conservation [2]. Montane habitats are characterized by mountainous regions coupled [3] with steep environmental gradients within small geographic areas and play a prominent role in shaping vertical species distributions due to the correlation between elevation and multiple environmental factors, such as temperature, humidity, and anthropogenic disturbances [1,4,5]. Among the environmental factors mentioned above, elevational gradients provide an excellent opportunity to assess biodiversity patterns and community structure [6]. Studies on elevational gradients focus on species diversity–altitude relationships in different taxa worldwide [4,7,8,9]. Despite their high sensitivity to changes in environmental factors [10], amphibians are the least studied taxonomic group. Additionally, previous studies tended to merely focus on medium–high elevations or are limited to small areas in mountainous regions [7,11,12]. For example, Khatiwada et al. [13] investigated the amphibian community structure along an elevational gradient of 78–4200 m in the eastern Himalayan region of Nepal, which showed a positive trend of decreasing amphibian species richness and abundance with increasing elevation in this region. Therefore, there is an apparent research gap on amphibian biodiversity in middle- and low-elevational areas and our study aims to provide greater information on these often-overlooked areas.

The theoretical frameworks of ecogeography can be used to characterize the effects of biodiversity on environmental stimuli [14]. Various ecogeographical rules (e.g., Rapoport’s rule, Gloger’s rule, and Bergmann’s rule) have been proposed to explain the responses of species to geographical distribution. Rapoport’s rule, which Stevens (1989) named and suggested, is one of them [15], and it has been expanded to applications on elevational gradients. It contends that species adapted to higher elevations have a wider distribution range because they are more climatically adaptable [13,16]. A variety of methods, such as Pagel’s method, Stevens’ method, mid-point method, and cross-species method, have been commonly used to evaluate Rapoport’s rule and give insights on various distribution patterns [17,18]. The first three methods mentioned above all determine the elevational Rapoport’s rule via analyzing the linear regression between mean species range size and elevation gradient. However, the cross-species method uses the elevation distribution of each species as an independent data point, allowing the incorporation of evolutionary data among the species during analysis [19]. Although each approach used to assess Rapoport’s rule has flaws of its own, they do establish a basic comprehension of elevational patterns in biodiversity that is nevertheless critical for conservation in interesting landmarks that are not as well documented.

To create effective conservation schemes and management strategies, it is important to know the various community structures that underpin and support the present biodiversity in the ecosystem [2]. Most studies focus only on the taxonomic element of species, regarding species as ecologically identical entities when analyzing how communities are formed [20]. Taxonomic diversity indices (e.g., species richness, Simpson index, and Shannon–Weiner index) reflect certain characteristics of species diversity to some extent, such as the abundance of species and the uniformity of their distribution [21]. However, the type of role and the magnitude of a species’ contribution to community construction is highly variable [21]. In the aforementioned indices, the removal of a single species does not greatly affect the results of the diversity index itself, but this could have significant ramifications in the community if they are considered to be keystone species [22]. Thus, it is crucial to determine the level of importance of certain species or taxa in biodiversity conservation to promote effective and targeted management policies [23]. 

Recent studies showed that there is a large gap between traditional biodiversity and practical conservation applications of biodiversity since the development of new metrics [24]. One of these new indices of species diversity is phylogenetic diversity which represents the sum of the phylogenetic branch lengths for all of the species in an area [23]. Species diversity reflects the level of species richness in a region, and the conservation of a species takes into account both the existing species richness and the evolutionary information it possesses [25,26]. Phylogenetic diversity is used to explain the evolutionary history of species from the perspective of kinship [25,27]. In recent years, with the combination of taxonomic and phylogenetic diversities [27,28], the inclusion of phylogenetic diversity has also been found to be more effective in revealing priority areas and taxa for biodiversity conservation when studying the diversity of animal taxa (e.g., amphibians and birds) compared to the single index in taxonomic diversity [29,30]. By utilizing a combination of diversity indices, this allows for a greater understanding of species dispersal, evolutionary history, and competitive ability in community structures, allowing biologists to better address key biodiversity trends [1,31]. Thus, combining phylogenetic and taxonomic diversities can provide new perspectives for biodiversity conservation.

According to the report of the International Union for Conservation of Nature (IUCN) [32], amphibians are declining more rapidly compared to mammals or birds. Currently, most extant amphibian species are categorized as threatened based on a global comparison and systematic assessment [33]. As ectotherms, the various life history of amphibians depends on various environmental factors, hence their responses to thermal changes in elevation, such as behavior and physiology, are tangible and palpable [34]. Species that are found in smaller ranges are more severely affected compared to species that are widely distributed due to their limited mobility [7]. Hence, amphibians provide an ideal system to explore the spatial and elevation patterns of animals and the potential factors that affect their richness for long-term biodiversity conservation. In this study, we investigated amphibian species diversity in the Fujian Junzifeng National Nature Reserve located in eastern China and tackled the subject of regional and elevational patterns of amphibian species diversity. We also examined the correlations within and between taxonomic and phylogenetic diversites, and finally, under the assumption that elevational Rapoport’s rule holds, the species range of amphibians in the nature reserve increases with elevation while also accounting for phylogenetic relationships.

## 2. Materials and Methods

### 2.1. Study Area

**Figure 1:**
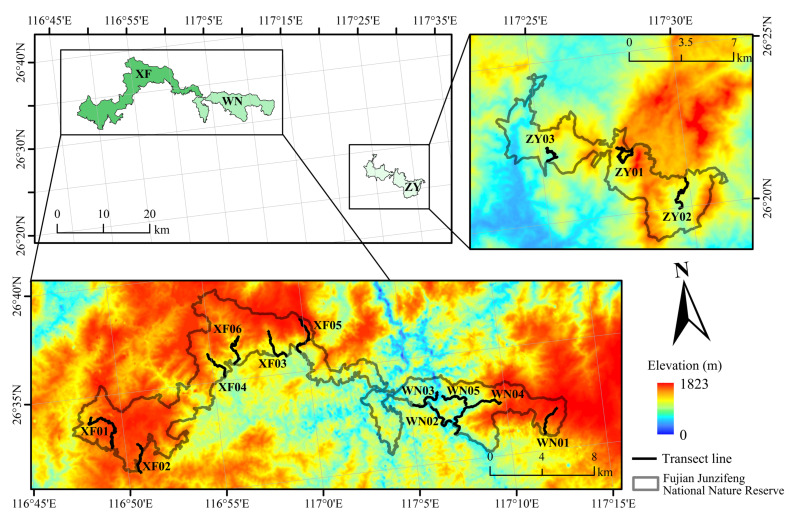
Sketch map of the transect lines in the Fujian Junzifeng National Nature Reserve in eastern China. XF: Xiafang district, WN: Wannei district, ZY: Ziyun district.

The study was conducted at Fujian Junzifeng National Nature Reserve (E116.7892°~E117.5228°, N26.3175°~N26.6550°) in eastern China, which is located in the subtropical region. Due to the large geographic area of the reserve, it is divided into three management districts, namely Xiafang (XF), Wannei (WN), and Ziyun (ZY). The reserve covers a total discontinuous area of 180.7 km^2^, containing a core zone of 75.0 km^2^, a buffer zone of 40.4 km^2^_,_ and an experimental zone of 65.3 km^2^ (Figure 1) [35]. Possessing an exceptionally rich amount of biodiversity, Fujian Junzifeng National Nature Reserve is home to over 472 species of wild vertebrates in 34 orders and 100 families due to its varied landscapes and complex habitats [35]. Coupled with a wide diversity of vegetation types and robust biodiversity, Fujian Junzifeng National Nature Reserve is considered to be one of the most unique ecological regions.

### 2.2. Survey Process

To understand the variability of biodiversity of amphibian species in the Fujian Junzifeng National Nature Reserve, 14 transect lines were set in the 3 districts (6 in XF, 5 in WN, and 3 in ZY, 3–5 people per field survey, and 6–12 days in each region) from April to August 2019 and 2020, focusing on elevation ranges from 210 m to 1251 m (Figure 1, Table S1) which were divided to 11 elevational sections of 100 m intervals each. The total length was 202 km (the specific length of each tansect is shown in Table S1), and the width of each transect line was 4 m. The survey was conducted between 19:00 h and 24:00 h in different habitats, such as streams, paddy fields, ponds, and roadside brush, and consisted of microhabitats, such as boulders, logs, mosses, and leaf litter, which were checked thoroughly for amphibians [7]. We recorded the number of each amphibian species seen along each transect line and their geographic information using GPS (eTrex 10, Garmin Ltd., Taiwan, China).

### 2.3. DNA Sequences Collection and Phylogenetic Tree Reconstruction

To determine the phylogenetic relationship of recorded amphibians in the nature reserve, we inventoried the surveyed amphibian species, downloaded the mitochondrial 12S (884 bp), 16S (1222 bp), and COI (1527 bp) sequence data of relevant species from GenBank (www.ncbi.nlm.nih.gov/genbank/, accessed on 1 January 2023) (Table S2), and performed sequence comparisons on MEGA X [36]. Concatenated sequence data of mitochondrial sequence data of the 29 amphibian species were used for phylogenetic reconstructions. A species-level phylogenetic tree was reconstructed using the Bayesian Inference (BI) method, conducted in MrBayes v. 3.2.7 [37], and selecting the GTR + I + G model, ran in jModeltest v. 2.1.4 [38] under the corrected Akaike’s Information Criterion (AIC) [39]. We used one *Ichthyophis bannanicus* sample as the outgroup. A dependent run with four chains was executed for 20 million generations with sampling every 1000 generations and the first 25% of trees were discarded as burn-in. The remaining trees were used to generate a consensus tree and calculate the posterior probabilities of all branches using a majority-rule consensus approach.

### 2.4. Species Diversity Metrics

Species diversity including taxonomic and phylogenetic diversities of amphibians was conducted in this study. Species richness (*S*), Simpson index (*C*), Shannon–Wiener index (*H’*), Pielou index (*E*), and Margalef index (*DMG*) were calculated for the taxonomic diversity [40,41] based on the following formulas, respectively:

*S* = number of species;
*C* = 1−$\sum P_{i}^{2}$;
*H’* = $-\sum_{i=1}^{S}P_{i}\;\ln P_{i}\;$;
*E* = *H’*/ln*S*;
*DMG* = (*S* – 1)/lnN;

where N is the total number of individuals, *P_i_* is an individual number of *i* species/total number of each sample.

Phylogenetic diversity was obtained via *picante* package in R 4.1.3. The sum of all branch lengths of the phylogenetic tree connecting all species in a local community was taken as Faith’s phylogenetic diversity index (*PD*) [23]. The phylogenetic structure is a complement to phylogenetic diversity and can reflect the ecological process of community construction [42]. The net relatedness index (*NRI*) focuses on the similarity of the phylogenetic relationship between species, and the nearest taxon index (*NTI*) focuses on the phylogenetic effect between similar species [43], thus these indexes were used as the phylogenetic structure [44]. The weight values of *NRI* and *NTI* were calculated using an independent swap null model with 999 randomizations and 1000 iterations based on the following formulas [44], respectively:

$$\mathrm{NRI}=1\times \frac{MPD_{obs}-\mathrm{mean}\left(MPD_{rnd}\right)}{sdMPD_{rnd}}$$

$$\mathrm{NTI}=-1\times \frac{MNTD_{obs}-\mathrm{mean}\left(MNTD_{rnd}\right)}{sdMNTD_{rnd}}$$

where *MPD_obs_* and *MNTD_obs_* are observations; *MPD_rnd_* and *MNTD_rnd_* represent the mean phylogenetic distance from 999 randomizations; *sd* is the standard deviation.

If *NRI* > 0 and *NTI* > 0, this indicates that the phylogenetic structure is clustered, and implies that the community is more likely to be composed of closely related species; if *NRI* < 0, *NTI* < 0, it suggests that the species community among species in this community tends to be more dispersed; if *NRI* = 0, *NTI* = 0, this indicates that the phylogenetic structure of the community is random.

### 2.5. Testing Elevational Rapoport’s Rule

To examine the elevational Rapoport’s rule, the cross-species method of Letcher and Harvey [19] was employed, which allows us to take into account phylogeny, and obtain the relationship between species range size and the corresponding midpoint of elevation after removing phylogenetic relationships. If they have a positive correlation, it supports the elevational Rapoport’s rule, otherwise, it is rejected [45].

### 2.6. Data Analyses

Before our analysis, we verified the normality and homogeneity of the data using Kolmogorov–Smirnov and Bartlett’s tests, respectively. Multiple comparisons were performed using Fisher’s Least Significant Difference (LSD) test. All values were presented as mean ± standard error (SE), and the differences were considered statistically significant at *p* < 0.05. One-way Analyses of Variances (ANOVAs) were used via STATISTICA v10.0 (Tulsa, OK, USA) to examine the differences in taxonomic and phylogenetic diversities including eight indexes among three districts and elevation gradients. The data of species richness and abundance from 14 transects in these 3 regions were used to calculate diversity indices and compare for regional species diversity divergence. In this survey, we were able to detect amphibians from 210 to 1250 m in the nature reserve (Figure S1). Despite the large surveyed range, we could only analyze the data within the 300–1000 m elevation range of the 3 districts. This is because we recorded amphibians below 300 m in only one transect in ZY, which was insufficient for us to run any robust analysis. At elevations above 1000 m, we only detected amphibians in the WN transect but were unable to do so in the same elevation for other XF transects despite records indicating the presence of amphibians at 1250 m in XF. Hence, our analysis only focuses on the elevation range between 300 and 1000 m based on data from three management districts despite our survey efforts. Pearson correlations were used via *corrplot* package [46] in R 4.1.3 to examine the relationship within and between taxonomic and phylogenetic diversities. Regression analyses with ordinary least squares (OLS) and phylogenetic general least squares (PGLS) methods were implemented via *rms* [47] and *caper* [48] packages in R 4.1.3 to examine the relationship between species range size and the corresponding midpoint of elevation of amphibians in the studied nature reserve. OLS model was used to estimate slopes for all conventional analyses. The PGLS model was used to examine the relationship while accounting for phylogeny and incorporates phylogenetic information into generalized linear models, which allowed us to analyze continuous data to estimate the evolutionary model and relationships among the traits of interest [49,50]. The likelihood-ratio test (LRT) based on AIC was used to assess the adequacy of the models used. Pagel’s lambda (λ) was used to measure the phylogenetic signal: the λ value of or near 1 indicates that the variable is fully explained by evolutionary history and indicates the strongest phylogenetic signal, and a value near 0 indicates phylogenetic independence [51].

## 3. Results

### 3.1. Species Composition and Their Phylogeny

Twenty-eight amphibian species belonging to eight genera, eight families, and two orders were recorded in the Fujian Junzifeng National Nature Reserve (Table 1). The most represented family was Ranidae, which includes 10 species, and they contributed approximately 35.7% of all recorded amphibian species in the nature reserve, followed by the family Dicroglossinae containing five species, and the family Salamandridae, which was represented by only one species (*Pachytriton brevipes*) (Table 1). Of the 28 species recorded, 1 species (*Hoplobatrachus chinensis*) was categorized as endangered, and 2 species (*Quasipaa exilispinosa and Q. spinosa*) were vulnerable according to China’s Red List of Biodiversity [52]. In addition, the families Salamandridae and Megophryidae included the newly discovered species *Boulenophrys sanmingensis* [53] and *Hyla sanchiangensis*, which were only observed in the WN district (Table 1). 

**Figure 2:**
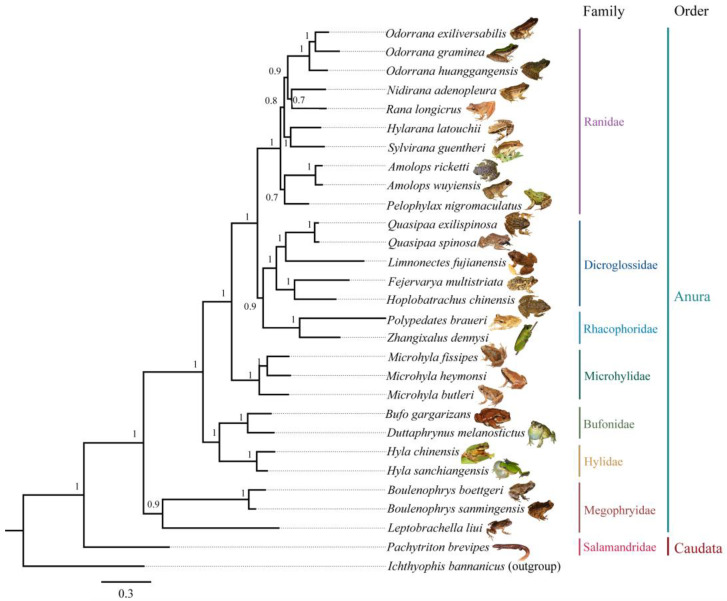
Phylogenetic relationship and elevational distribution for amphibian species in the Fujian Junzifeng National Nature Reserve in eastern China. The tree was reconstructed using Bayes Interfere method based on mitochondrial 12S + 16S + CO1 gene sequences (3633 bp). Bayesian posterior probability of each node are shown with maxima of 1.00. The accession numbers of each gene are shown in Table S2. Photographs were taken by Guo-Hua Ding.

The phylogenetic relationships constructed for the 28 amphibian species recorded in this study show that (1) *P. brevipes* was located at the root of the evolutionary tree; (2) the genus *Boulenophrys* and *Leptobrachella* formed a sister group sister, and they were a basal clade relative to others within the order Anura; and (3) the family Rhacophoridae formed a sister group with the family Dicroglossinae, and they in turn were a sister group with the family Ranidae (Figure 2).

### 3.2. Regional Taxonomic and Phylogenetic Diversities

**Figure 3:**
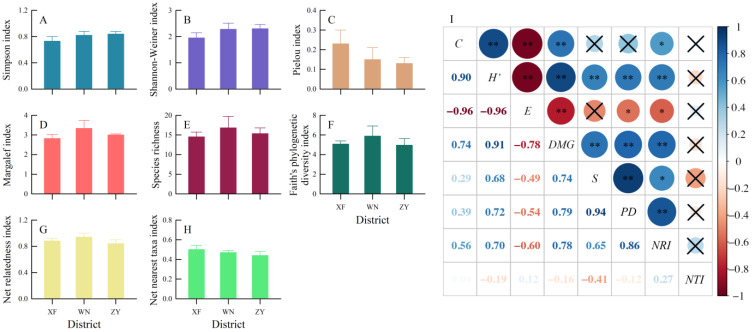
Mean (+SE) for Simpson index (**A**), Shannnon-Weiner index (**B**), Pielou index (**C**), Margalef index (**D**), Species richness (**E**), Faith’s phylogenetic diversity index (**F**), net relatedness index (**G**), net nearest index (**H**) among the three regions, and their Pearson correlation for regional scale (**I**) in the Fujian Junzifeng National Nature Reserve in eastern China. XF: Xiafang district, WN: Wannei district, ZY: Ziyun district. *C*: Simpson index, *H’*: Shannon-Weiner index, *E*: Pielou index, *DMG*: Margalef index, *S*: species richness, *PD*: Faith’s phylogenetic diversity index, *NRI*: net relatedness index, *NTI*: net nearest taxa index. The color gradient from red to blue in figure I represents the correlation coefficient ranging from −1 to 1. The symbol “×” indicates no correlation between the corresponding pairs of indices. *: *p* < 0.05, **: *p* < 0.01.

Mean values for five taxonomic diversity indexes (*C*, *H’*, *E*, *DMG*, and *S*) and three phylogenetic diversity indexes (*PD*, *NRI*, and *NTI*) did not differ among the three districts (all *p* > 0.4; Figure 3A–H). The results of the Pearson correlation showed that (1) there is a correlation within species diversity for all except *C* and *S* (both *p* > 0.05), *C, H’,* and *DMG* were negatively correlated with *E* (all *p* < 0.01), and there was a positive correlation between the other two indexes (all *p* < 0.01); (2) within phylogenetic diversity, there was a positive correlation between *PD* and *NRI* (*p* < 0.01); (3) between taxonomic and phylogenetic diversities, *PD* and *NRI* were positively correlated with *H’*, *DMG,* and *S*, while there is a negative association with *E* (all *p* < 0.05); additionally, *NRI* was positively correlated with *C* (*p* < 0.05) (Figure 3I).

### 3.3. Elevational Taxonomic and Phylogenetic Diversities

**Figure 4:**
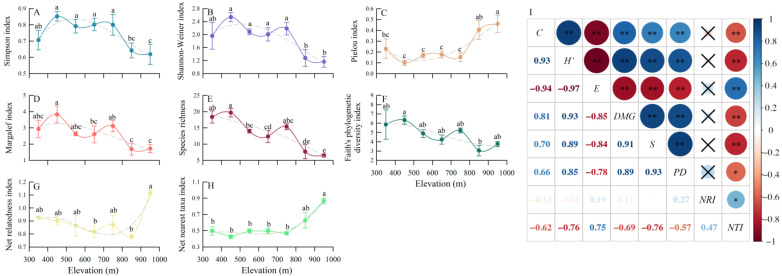
Mean (±SE) for Simpson index (**A**), Shannnon-Weiner index (**B**), Pielou index (**C**), Margalef index (**D**), Species richness (**E**), Faith’s phylogenetic diversity index (**F**), net relatedness index (**G**), net nearest index (**H**) along the elevational gradient, and their Pearson correlation for elevational scale (**I**) in the Fujian Junzifeng National Nature Reserve in eastern China. Different alphabets above the dots indicate significant difference (Fishers LSD test, α = 0.05, a > b > c > d > e). Regression curves are represented by dash lines, and their corresponding regression equations and coefficients can be found in Table S3. *C*: Simpson index, *H’*: Shannon-Weiner index, *E*: Pielou index, *DMG*: Margalef index, *S*: species richness, *PD*: Faith’s phylogenetic diversity index, *NRI*: net relatedness index, *NTI*: net nearest taxa index. The color gradient from red to blue in figure I represents the correlation coefficient ranging from −1 to 1. The symbol “×” indicates no correlation between the corresponding pairs of indices. *: *p* < 0.05, **: *p* < 0.01.

Mean values for five taxonomic diversity indexes (*C*, *H′*, *E*, *DMG*, and *S*) and three phylogenetic diversity indexes (*PD*, *NRI*, and *NTI*) differed significantly among elevations (all *p* < 0.05). The elevational pattern showed that (1) except for *E*, the other four taxonomic diversity indices had a similar hump-shaped pattern; (2) the mean values for *C*, *H’*, *DMG*, and *S* decreased sharply when the elevation ranged from 800 m to 1000 m, whereas the mean *E* increased (Figure 4A–E); (3) the mean *PD* decreased gradually with the increase in elevation (Figure 4F); (4) the mean *NRI* and *NTI* gradually increased at the elevations below 900 m, but sharply increased at the elevation of 900–1000 m (Figure 3G,H). The results of the Pearson correlation showed that in the elevation scale, (1) *NRI* was positively related to *NTI* (*p* < 0.05), but not with the other indexes (all *p* > 0.05); (2) *E* was positively correlated with *NTI* but was negatively correlated with other indexes (all *p* < 0.01); (3) *NTI* was negatively related with the remaining species diversity indexes (all *p* < 0.01); and (4) there was a positive correlation between *NTI* and *PD* (*p* < 0.05) (Figure 4I).

### 3.4. Elevational Rapoport’s Rule

**Figure 5:**
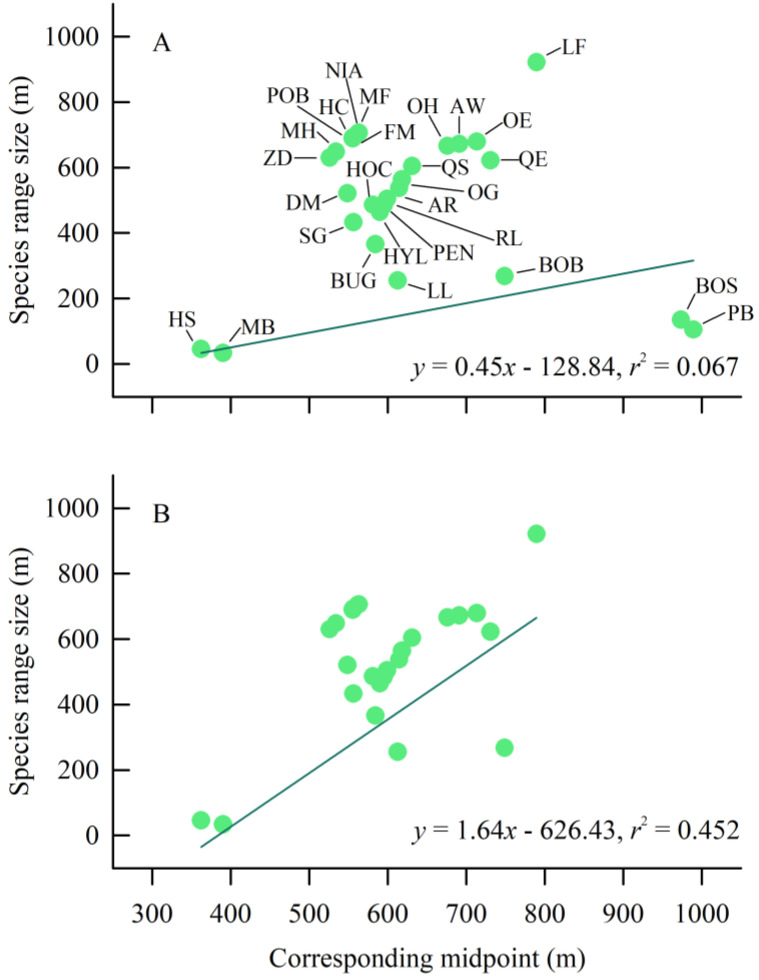
The relationship between species range size and corresponding midpoint of altitude to the results of the cross-species method of Letcher and Harvey for the surveyed amphibians in the Fujian Junzifeng National Nature Reserve in eastern China. (**A**): the data of all species were used; (**B**): the data of the species (BOS and PB) observed only in one transect line were deleted. Regression equations and coefficients based on phylogenetic generalized least squares (PGLS) method are shown in the figure. Species abbreviations are shown in Table 1.

The PGLS analysis showed that a strong phylogenetic signal affected the species range size and the corresponding midpoint of amphibians (λ > 0.7, Table 2). In both OLS and PGLS models, no significant correlation emerged between amphibian species range size and the corresponding midpoint of elevation when the whole sampling dataset was processed (*p* > 0.05; Figure 5A). However, species range size was positively correlated with the corresponding midpoint of elevation only when the cross-species method was employed to analyze parts of the sampling dataset which excludes the species (*P. brevipes* and *B. sanmingensis*) observed only in one transect line (*p* < 0.001; Figure 5B). Based on LRT, the PGLS model was a better fit for the data than the OLS model for species range size versus the corresponding midpoint of elevation (Table 2).

## 4. Discussion

In this study, two dimensions of species diversity (taxonomic and phylogenetic diversities) were combined to analyze spatial (regional and elevational) divergence in amphibian diversity in the Fujian Junzifeng National Nature Reserve, eastern China. Our results revealed that (1) most of the taxonomic and phylogenetic diversity metrics were correlated; for regional divergence, *PD* and *NRI* were positively correlated to *H’*, *DMG,* and *S*, while negatively with *E*; for elevational divergence, only *E* was positively correlated to *NTI*, but was negatively correlated with other indices. These findings emphasize the significance of a multi-dimensional biodiversity study and urge caution when substituting one diversity index component for another. (2) The mean values for all diversity indices did not differ among the three districts but differed significantly along the elevational gradient. (3) The species range size and the corresponding midpoint of amphibians were affected by a strong phylogenetic signal, which supported the elevational Rapoport’s rule after removing the data of *P. brevipes* and *B. sanmingensis*. 

The small populations of these two species were discovered only in a transect above 900 m in elevation. *P. brevipes* are usually detected in steeper brooks in mountainous areas from the elevation of 800–1700 m [54], and most of the transects in our survey were distributed at lower elevational zones. In the higher elevation of the XF region, there are fewer creeks as most of them have been dried up, resulting in the loss of suitable habitats for *P. brevipes*. Meanwhile, *B. sanmingensis* is a new species discovered in 2021, and it is found in very limited areas [53]. Based on both species’ preferred habitat for moist and cool environments [54], we suggest that we extend our survey range area in future studies. In light of their rarity and limited distribution, we believe appropriate conservation measures are necessary for this area as there is still a dearth of understanding of the basic ecological roles or behavior of these two species. In addition, future research could consider a larger elevation gradient and geographic area to encompass more organisms. Altogether, these findings could contribute to a better understanding and more effective conservation of the mountain’s amphibian diversity.

### 4.1. Regional Species Diversity

Biodiversity is the basis of regional sustainable development. An accurate understanding of biodiversity within a region is of great significance in reflecting the status quo, changes, and threats to regional ecosystems, as well as selecting decisive conservation strategies [55]. By comparing species composition and patterns of species richness across regions, the focus of the study is to resolve the composition of existing species diversity [56]. In this study, we did not find significant divergence in the taxonomic and phylogenetic diversities across the three management units (XF, WN, and ZY), indicating that the species composition and richness of the three regions are somewhat similar. 

Although it has been demonstrated that longitude and latitude are significant spatial factors that impact the population of aquatic organisms, the primary drivers of species diversity are various local environmental factors [57]. For example, the species richness of amphibians diminishes with increasing latitude in the southwest karst landscape, but this trend is primarily the product of the interaction between geomorphology and several environmental conditions [58]. In the study, the three regions are similar in latitude, and although there are slight differences in longitude, these regions are approximately similar with a consistent macroclimate [35]. Additionally, longitude may affect the variation of vegetation types [59], and during the survey, we discovered that the vegetation types in all three regions are comparable and primarily comprise deciduous broadleaf forest, mixed evergreen deciduous broadleaf forest, evergreen broadleaf forest, bamboo forest, evergreen broadleaf scrub and grass, and agricultural land. The same composition of vegetation types constitutes a similar habitat, which may also explain the similarity of amphibian species diversity in the three regions [35]. We speculate that the three regions’ identical climates, comparable precipitation, and geographic proximity may be to blame for the lack of distinctions in amphibian species at the longitude level. Therefore, long-term monitoring and large-scale surveys are necessary for a more in-depth and accurate understanding of amphibian diversity.

### 4.2. Elevational Pattern

Biodiversity research on elevational gradients has provided insights into the spatial distribution of organisms [60]. In this study, we observed that the four indices of taxonomic diversity (*C*, *H’*, *DMG*, and *S*) showed the hump-shaped pattern along the whole elevational gradients in the Junzifeng Nature Reserve which featured high taxonomic diversity between 300 and 800 m. Previous studies have also confirmed that amphibian species richness exhibited a hump-shape response to elevational gradients in other mountains, such as the Hengduan Mountains [45] and Qinling Mountains [61] in China, and the tropical Andes on the west coast of South America [62]. This pattern occurred mainly due to limited sampling effort, constraints on species range boundaries, and geographical scales [13,63]. It is generally accepted that lower elevations have higher temperatures and precipitation, creating an environment that is more suited for many ectotherms, such as amphibians, and may sustain a broader variety of species and individuals [4,6,13,64]. At relatively higher elevations, there were drier streams in the nature reserve, and we speculate that this led to a decline in amphibian species that preferred wetter habitats, resulting in lower species diversity at higher elevations. This is probably one of the more significant reasons for the emergence of taxonomically diverse elevational patterns in our current research. Additionally, our results were congruent with the findings of Chen et al. [65] and Wang et al. [66], which could be attributed to the similar climatic conditions and vegetation types of the reserve located on the periphery of Wuyi Mountains in eastern China. 

The variations of phylogenetic diversity with elevations were variable across different taxa in different mountains [60]. In our studied nature reserve, the *PD* values of amphibians also showed a hump-shaped relationship with elevation, which was similar to other reported mountainous areas, such as the eastern Himalayas of Nepal [20]. Interestingly, the elevations of 300–500 m seemed to have the maximum *PD* value, which could be explained by the presence of evolutionary key species which are narrowly distributed along low elevations [20]. *PD* is typically positively connected with species richness, which illustrates why its values were considerably stronger in low elevations than in high elevations [20]. However, this observation is contrasted with the previous study where the *PD* values of amphibians showed a unimodal variation with altitude in montane ecosystems located at the eastern margin of the Qinghai–Tibetan Plateau [6]. In addition, the *PD* values vary with elevation across different taxa in different mountains, with declining [67,68], hump-shaped [69], and increasing patterns detected [70]. Overall, these various phylogenetic patterns reflected the adaptation or non-adaptation of species to the mountainous landscape in terms of elevational distribution, thus showing the specificity of each mountain ecosystem, resulting in different vertical distribution patterns.

Based on the premise of phylogenetic ecological niche conservatism [71], the divergence of phylogenetic structure can indicate that competition plays a dominant role among species distribution, whereas phylogenetic clustering showed that collaboration plays a more dominant role [72,73]. In this study, the *NTI* and *NRI* for the phylogeny showed similar elevational patterns, where the NRI and NTI values for each elevation gradient were above zero, showing a clustered phylogenetic structure similar to previous findings in plants [74] and tropical mountain birds [68]. This result may be explained by the vertical geographic effect acting as a significant filter, removing specialized taxa and phylogenetically distant groups and leaving more generalists and closely related species [1,75,76]. Harsh climatic or damp environments were frequently considered to be ecological filters for amphibians’ distribution, and species that can withstand harsh environments tend to be more favored [69]. Generally, the aggregation extent decreases with increasing elevation, but a contrary result was obtained in our study as our *NTI* and *NRI* values abruptly steepened (*NRI* > 1 and *NTI* converges to 1) in the elevations of 900–1000 m, which showed a strict agglomeration pattern. It could possibly be due to how fewer amphibian species are dispersed at this range of elevation, and they have relatively close evolutionary relationships. By combining the *NTI* and *NRI* results from this study, it seems that the community dynamics across the closest kinship dominate the entire phylogenetic structure [75]. In addition, limited resources encourage amphibians to specialize in ecological niches along with decreasing habitat areas and other resources [6,20,70]. 

### 4.3. Elevational Rapoport’s Rule Examination

Closely related species tend to have similar resource requirements from the environment and competitive exclusion can limit the coexistence of closely related species [77]. The species range is the maximum area and extent over which a species can be distributed spatially. It influences species differentiation and extinction and is an important ecological and evolutionary feature of species [16,64]. In our study, the PGLS analysis showed that the strong phylogenetic signal affected species range sizes and corresponding midpoints in amphibians.

The elevational Rapoport’s rule has been considered as one of the key assumptions on vertical distribution patterns associated with species richness [78]. However, previous studies showed that Rapoport’s rule did not apply to amphibians on a global scale [16,79,80]. Our data showed that the cross-species method validated the rule only when the influence of the low-frequency occurrence probability of the surveyed species (e.g., family Salamandridae) was excluded, which is congruent with the findings of Chen et al. [65]. The rule also states that species adapted to higher altitudes should have a larger distribution range due to climate tolerance [81]. Considering the differences in the complexity of factors and the temperatures in the habitats at varying altitudes, species found at higher altitudes have higher adaptability to extreme environments and are more widely distributed, compared to those that dwell at lower elevations [65]. Overall, these results indicate that amphibians distributed at higher elevations in the mountains of eastern China may have a species range size broader than those at lower elevations. Therefore, understanding the key determinants of species range size would aid in explaining global ecological patterns and estimating the extinction risk of species [16].

### 4.4. Limitation

Although we used taxonomic and phylogenetic diversities, other dimensions of diversity (e.g., functional diversity, and phylogenetic beta diversity) and environmental factors could be considered for future studies for a greater understanding of the elevational distribution of amphibians. Additionally, we did not yield sightings of the Chinese giant salamander *Andrias davidianus* during our survey, which is a Class II nationally protected species with extremely low populations in China. Its rarity might be one reason that we failed to detect them in this study. The family Salamandridae was observed only on one transect line in the WN district, but it may be distributed elsewhere, perhaps owing to a lack of survey experience and limitations in survey coverage resulting in a scarcity of data. Therefore, long-term monitoring in the field and expansion of monitoring coverage are needed. For future studies, we will aim to improve our experimental methods by training surveyors for more comprehensive surveys, increasing our survey range, as well as integrating multiple aspects for our analysis. In summary, the results of this study serve as a baseline for future studies in the distribution mechanism of amphibian diversity along elevational gradients using a combination of diversity indices.

## 5. Conclusions

From our study results, we obtained the following conclusions. Firstly, at the regional level in the Fujian Junzifeng National Nature Reserve, positive correlations were observed between *PD* and *NRI* with *H’*, *DMG*, and *S*, while negative correlations were noted with *E*; at the elevational level, a positive correlation was found between *E* and *NTI*, but negative correlations were evident for the other indices. Secondly, no significant differences were found in measured taxonomic and phylogenetic diversity indices among the three management units. However, we found significant variations along the elevational gradient in the nature reserve of these indices. Lastly, after excluding data on *P. brevipes* and *B. sanmingensis* in the nature reserve, the elevational Rapoport’s rule could be supported using the cross-species method. Therefore, these findings could potentially contribute towards a better understanding and more effective conservation of amphibian species diversity in the mountainous regions of eastern China.

## Figures and Tables

**Table 1:** List of recorded amphibians in Fujian Junzifeng National Nature Reserve. Conservation status, altitude range, and survey region are shown in the table. LC: least concern, NT: near threatened, VU: vulnerable, EN: endangered, NE: not evaluated; XF: Xiafang district, WN: Wannei district, ZY: Ziyun district; ●: recorded.

Family	Species	Species Abbreviation	Conservation Status	Elevation Range (m)	Management District		
					XF	WN	ZY
Salamandridae	*Pachytriton brevipes*	PB	LC	937−1042		●	
Megophryidae	*Leptobrachella liui*	LL	LC	485−740		●	
	*Boulenophrys* *boettgeri*	BOB	LC	615−883		●	
	*Boulenophrys* *sanmingensis*	BOS	NE	906−1041		●	
Bufonidae	*Bufo gargarizans*	BUG	LC	401−767	●	●	●
	*Duttaphrynus melanostictus*	DM	LC	288−809	●	●	●
Hylidae	*Hyla chinensis*	HC	LC	210−901	●	●	●
	*Hyla sanchiangensis*	HS	LC	339−385		●	
Microhylidae	*Microhyla butleri*	MB	LC	373−407		●	●
	*Microhyla fissipes*	MF	LC	210−916	●	●	●
	*Microhyla heymonsi*	MH	LC	210−858	●	●	●
Dicroglossinae	*Fejervarya multistriata*	FM	LC	210−902	●	●	●
	*Hoplobatrachus chinensis*	HOC	EN	338−824	●	●	●
	*Limnonectes fujianensis*	LF	NT	329−1250	●	●	●
	*Quasipaa exilispinosa*	QE	VU	420−1042	●	●	●
	*Quasipaa spinosa*	QS	VU	329−933	●	●	●
Ranidae	*Amolops ricketti*	AR	LC	346−883	●	●	●
	*Amolops wuyiensis*	AW	LC	355−1027	●	●	●
	*Sylvirana guentheri*	SG	LC	340−773	●	●	●
	*Hylarana latouchii*	HYL	LC	358−822	●	●	●
	*Nidirana adenopleura*	NIA	LC	210−914	●	●	●
	*Odorrana exiliversabilis*	OE	LC	374−1053		●	●
	*Odorrana huanggangensis*	OH	LC	343−1009	●	●	●
	*Odorrana graminea*	OG	NT	336−900	●	●	●
	*Pelophylax nigromaculatus*	PEN	NT	353−837	●	●	
	*Rana longicrus*	RL	LC	347−851	●	●	●
Rhacophoridae	*Polypedates braueri*	POB	LC	211−900	●	●	●
	*Zhangixalus dennysi*	ZD	LC	211−841	●	●	●

**Table 2:** Regressions between species range size and corresponding midpoint of altitude of recorded amphibians in the Fujian Junzifeng National Nature Reserve based on ordinary least squares (OLS) and phylogenetic generalized least squares (PGLS) methods. *: On the basis of likelihood ratio test, the model which is labeled statistically significant is better than the OLS model.

	Data of All the Species		Removal of the Species Observed Only in One Transect Line	
	OLS Model	PGLS Model	OLS Model	PGLS Model
*N*	28	28	26	26
Slope	−0.06 ± 0.32	0.45 ± 0.33	1.17 ± 0.37	1.64 ± 0.37
Intercept	542.76 ± 206.56	−128.84 ± 292.98	−151.10 ± 223.31	−626.43 ± 245.72
*r* ^2^	0.001	0.067	0.293	0.452
Ln likelihood	−190.986	−187.074 *	−170.408	−167.876 *
AIC	387.972	382.147	346.816	343.752
λ	–	0.707 (0.194–0.983)	–	0.775 (0.070-NA)
Statistical results	*F*_1, 26_ = 0.04, *p* = 0.851	*F*_1, 26_ = 1.88, *p* = 0.182	*F*_1, 24_ = 9.93, *p* < 0.01	*F*_1, 24_ = 197.77, *p* < 0.001

## Data Availability

The data presented in this study are openly available in ScienceDB (Science data bank) (https://doi.org/10.57760/sciencedb.06422). Further inquiries can be directed to the corresponding author.

## Supplementary Materials

The following supporting information can be downloaded at https://www.mdpi.com/article/10.3390/biology12030461/s1: Table S1: The transect lines of amphibian monitoring in the Fujian Junzifeng National Nature Reserve.; Table S2: GenBank accession numbers for amphibian species used in the phylogenetic analyses.; Table S3: Regression equations and coefficients between diversity indices and elevation.
