# Supplementary material for: Spatial Patterns of Species Diversity of Amphibians in a Nature Reserve in Eastern China
Source: Biology (Basel). 2023 Mar 16;12(3):461. doi: 10.3390/biology12030461 (PMC10045056; doi:10.3390/biology12030461)
Supplement: Supplementary file 1 [file biology-12-00461-s001.zip › biology-2112763-SI.pdf]

Spatial Patterns of Species Diversity of Amphibians in a Nature Reserve from Eastern China

Yan-Mei Wang, Hua-Li Hu, Lei Feng, Jing-Yi Chen, Jun-Jie Zhong, Rachel Wan Xin Seah, Guo-Hua Ding

Supplemental materials

Table S1. The transect lines of amphibian monitoring in the Fujian Junzifeng National Nature Reverse from eastern China.

Mangement district	Area (km^2)	Tracset line ID	Length (km)	Longitude and latitude (°)		Elevational range (m)
				Starting	Ending	
Xiafang district (XF)	93.3	XF01	8.14	E116.81, N26.56	E116.83, N26.54	601-1,796
		XF02	3.51	E116.85, N26.54	E116.85, N26.52	650-796
		XF03	3.76	E116.97, N26.61	E116.99, N26.59	427-647
		XF04	2.94	E116.92, N26.60	E116.93, N26.58	430-528
		XF05	5.25	E117.00, N26.62	E117.00, N26.59	430-1,251
		XF06	6.72	E116.95, N26.61	E116.94, N26.59	429-614
Wannei district (WN)	50	WN01	5.50	E117.20, N26.51	E117.22, N26.53	547-1,056
		WN02	3.60	E117.11, N26.54	E117.12, N26.53	304-361
		WN03	4.18	E117.09, N26.54	E117.12, N26.55	332-383
		WN04	7.39	E117.17, N26.54	E117.12, N26.52	343-596
		WN05	4.92	E117.14, N26.54	E117.12, N26.55	310-407
Ziyun district (ZY)	31.3	ZY01	4.58	E117.48, N26.36	E117.47, N26.36	563-849
		ZY02	3.69	E117.50, N26.33	E117.50, N26.35	453-725
		ZY03	3.86	E117.41, N26.35	E117.43, N26.36	210-489

Table S2. GenBank accession numbers for amphibian species used in the phylogenetic analyses.

ID	Species	Mitochondrial DNA		
		12S	16S	CO1
01	*Pachytriton brevipes*	NC_053711	NC_053711	NC_053711
02	*Leptobrachella liui*	MH406642	MH923370	MH406371
03	*Boulenophrys boettgeri*	MH406519	MH406695	MH406143
04	*Boulenophrys sanmingensis*	–	MH406697	MH406145
05	*Bufo gargarizans*	NC_008410	NC_008410	NC_008410
06	*Duttaphrynus melanostictus*	NC_005794	NC_005794	NC_005794
07	*Hyla chinensis*	NC_006403	NC_006403	NC_006403
08	*Hyla sanchiangensis*	MZ508281	MZ508281	MZ508281
09	*Microhyla butleri*	NC_030049	NC_030049	NC_030049
10	*Microhyla fissipes*	NC_045110	NC_045110	NC_045110
11	*Microhyla heymonsi*	NC_006406	NC_006406	NC_006406
12	*Fejervarya multistriata*	NC_029754	NC_029754	NC_029754
13	*Hoplobatrachus chinensis*	NC_019615	NC_019615	NC_019615
14	*Limnonectes fujianensis*	NC_007440	NC_007440	NC_007440
15	*Quasipaa spinosa*	NC_013270	NC_013270	NC_013270
16	*Quasipaa exilispinosa*	NC_056269	NC_056269	NC_056269
17	*Amolops ricketti*	NC_023949	NC_023949	NC_023949
18	*Amolops wuyiensis*	NC_025591	NC_025591	NC_025591
19	*Sylvirana guentheri*	MN248533	MN248533	MN248533
20	*Hylarana latouchii*	NC_057198	NC_057198	NC_057198
21	*Nidirana adenopleura*	NC_018771	NC_018771	NC_018771
22	*Odorrana graminea*	NC_050884	NC_050884	NC_050884
23	*Odorrana huanggangensis*	MK650099	KF185059	–
24	*Odorrana exiliversabilis*	NC_053712	KF185056	NC_053712
25	*Pelophylax nigromaculatus*	KT878718	KT878718	KT878718
26	*Rana longicrus*	MZ680528	MZ680528	MZ680528
27	*Polypedates braueri*	NC_042797	NC_042797	NC_042797
28	*Zhangixalus dennysi*	NC_027452	NC_027452	NC_027452
29	*Ichthyophis bannanicus* (outgroup)	NC_006404	NC_006404	NC_006404

Table S3. Regression equations and coefficients between diversity indices and elevation.

Diversity index	Regression equation	Regression coefficient
Simpson index	$y = -0.445 + 0.0057x - 7.80*10^{-6}x^2 + 3.17*10^{-9}x^3$	0.84
Shannon-Wiener index	$y = -1.687 + 0.0184x - 2.59*10^{-5}x^2 + 1.01*10^{-8}x^3$	0.80
Pielou index	$y = 1.114 - 0.0042x + 5.19*10^{-6}x^2 - 1.58*10^{-9}x^3$	0.87
Margalef index	$y = -0.453 + 0.0189x - 2.93*10^{-5}x^2 + 1.23*10^{-8}x^3$	0.65
Species richness	$y = 28.34 - 0.0392x + 4.75*10^{-5}x^2 - 3.24*10^{-8}x^3$	0.82
Faith's phylogenetic diversity index	y = 2.61 + 0.0227x - 4.52*10-5x2 + 2.37*10-8x3	0.71
Net relatedness index	$y = -0.2989 + 0.0074x - 1.42*10^{-5}x^2 + 8.33*10^{-9}x^3$	0.78
Nearest taxon index	$y = -0.1825 + 0.0042x - 8.55*10^{-6}x^2 + 5.57*10^{-9}x^3$	0.95
